# Important discoveries from analysing bacterial phenotypes

**DOI:** 10.1111/j.1365-2958.2008.06383.x

**Published:** 2008-10

**Authors:** Barry R Bochner, Luciana Giovannetti, Carlo Viti

**Affiliations:** 1 Biolog, Inc.21124 Cabot Blvd., Hayward, CA 94545, USA; 2 Dipartimento di Biotecnologie Agrarie, Università degli Studi di FirenzeFlorence 50144, Italy

## Abstract

The ability to test hundreds to thousands of cellular phenotypes in a single experiment has opened up new avenues of investigation and exploration and led to important discoveries in very diverse applications of microbiological research and development. The information provided by global phenotyping is complementary to, and often more easily interpretable than information provided by global molecular analytical methods such as gene chips and proteomics. This report summarizes advances presented by scientists brought together to share their experiences and knowledge gained with high-throughput phenotyping.

## Conference on bacterial phenotyping

A conference held in Florence, Italy, 19–21 March, chaired by Dr. Carlo Viti of the Università degli Studi di Firenze, brought together microbiologists using a new high-throughput phenotyping technology to study diverse problems in bacteriology. The meeting covered a broad range of topics as indicated by its title, ‘Florence Conference on Phenotype MicroArray Analysis of Microorganisms: The Environment, Agriculture, and Human Health’. There were many interesting and exciting presentations, some of which could be considered as major breakthroughs. Common to all of these presentations was the use of a high-throughput cell-phenotyping method to obtain information that has not been accessible using other methods available to microbiologists. This technology and its proposed importance were last reviewed in 2003 (Bochner, 2003).

## Studies of important bacteria that are difficult to culture: mycobacteria

Many microbial cells remain unculturable or very difficult and slow to culture. When these microorganisms are important pathogens, this slows the progress in studying them and hinders the entire process of finding curative treatments. Phenotype MicroArray (PM) technology provides a set of nearly 2000 culture conditions in which one can test the ability of a microorganism to respire and grow. The set includes about 200 C-sources, 400 N-sources, 100 P- and S-sources, 100 nutrient supplements, and a range of conditions varying the pH, ion and osmotic status of the culture environment. This enables scientists to see what stimulates growth and, equally important, what inhibits growth. The technology was first developed for *Escherichia coli* (Bochner *et al*., 2001; Zhou *et al*., 2003) but there are now protocols for testing of more than 1000 bacterial species, as well as most yeast and filamentous fungi.

The Pasteur and Russian strains of *Mycobacterium bovis* BCG have a long and important history in medical microbiology as general immunological adjuvants, and as attenuated vaccine strains for tuberculosis, which caused 1.7 million deaths in 2006. The Pasteur strain is a low producer of the key antigen protein mpb70, whereas the Russian strain is a high producer. Both strains grow very slowly. With a doubling time of about 24 h, they take about 2 weeks to culture in liquid and 4–8 weeks to form colonies on the most optimal agar culture media. Furthermore, their metabolic properties have remained refractory to study. Bhagwati Upadhyay and Paul Wheeler (VLA, Weybridge, UK) reported the successful development of a PM protocol for testing strains of *M. bovis* in spite of difficulties in consistent elimination of background metabolism. The PM assay required only a 5-day incubation. Both strains could metabolize glucose, pyruvate, glycerol, dihydroxyacetone, tweens and methyl-succinate, but there were also several differences that distinguished the strains, both in carbon metabolism (for d-lactose, cellobiose, gentiobiose, amygdalin, salicin, l-asparagine, d-alanine, l-alanyl-glycine, fumaric acid and bromo-succinic acid) and in nitrogen metabolism (l-glutamine). These differences, together with the difference in mpb70 secretion, have arisen over about 36 years of subculturing encompassing ∼800 passages (Brosch *et al*., 2007). The culture medium employed contained glycerol and starch, neither of which could be used by the *M. bovis* isolate originally selected for attenuation (Keating *et al*., 2005). Thus, with the severe selection pressure under which the bacteria were placed to enable them to grow on glycolytic carbon sources, a wide range of changes in both metabolism and antigen production appear to have occurred, presumably by mutation. The challenge going forward will be to determine if there is a causal link between metabolism and antigenicity or whether these changes occurred independently. This work provides not only the first detailed metabolic characterization of these strains, but it may lead to more rapid diagnostic capabilities as well as better methods for culturing and isolating *M. bovis*.

## Studies of pathogenic bacteria growing inside and alongside animal cells

Also in the category of important bacteria that are difficult to culture are those that survive and grow inside animal cells. *Coxiella burnetii*, the agent of Q-fever, is an obligate intracellular parasitic organism for which axenic (host cell-free) culture conditions have never been established. This bacterium replicates in lysosomal-like vacuoles of phagocytes at pH 4.5–5.0, but it has not been possible to reproduce the essential aspects of this intracellular environment and culture the bacteria when they are removed from their host cells. However, Anders Omsland (NIH, Rocky Mountain Laboratories, Hamilton, Montana) reported work with his colleague, Robert Heinzen, employing PM technology to establish *in vitro* culture conditions that allowed the metabolic properties of this bacterium to be studied. They were successful because the standard PM technology measures cell respiration and does not require that the cells grow. Therefore, the metabolism of *C. burnetii* recovered from eukaryotic host cells, following mechanical disruption of the host cells, could be assayed in PM panels to define which nutrients it relies on, or is inhibited by, during host cell-free metabolism. Anders reported that *C. burnetii* utilized 17 of the 95 C-source substrates in the C-source assay panel PM1, whereas other substrates had adverse effects on metabolic activity. Metabolism was maximal under microaerobic conditions (2.5% oxygen) and acid pH (4.5). Using this information about the physiology of *C. burnetii*, Anders has developed a Complex Coxiella Medium (CCM) and is now able to keep these bacteria metabolically active in a cell-free environment, for more than 24 h, allowing him to use pulse labelling with radioactive isotopes to study *de novo* protein synthesis in a cell-free environment. In addition, PM technology will allow high-throughput validation of *C. burnetii* nutrient utilization based on *in silico* metabolic pathway reconstruction.

In a somewhat similar fashion, *Legionella pneumophila* infects and grows inside alveolar macrophages and causes a life-threatening pneumonia. Inside the macrophage, it goes from a transmissive form to a non-motile and non-infectious replicative form and then back to a non-replicative transmissive form (Sauer *et al*., 2005). Rachel Edwards from Michele Swanson's laboratory (University of Michigan, Ann Arbor) used PM technology to screen a strain carrying a *flaA–gfp* fusion (an indicator of differentiation to a motile state) under hundreds of culture conditions, and discovered that growth arrest and the transition back to the transmissive form are triggered by carboxylic acids, especially the short-chain fatty acids acetic and propionic acid. Moreover, the ability of *L. pneumophila* to sense the short-chain fatty acids was dependent on the LetA/LetS two-component system and the stringent response enzyme SpoT. They predict that SpoT likely interacts with acyl carrier protein to monitor flux in fatty acid biosynthesis and regulate its phase differentiation.

Barry Bochner (Biolog, Hayward, CA) presented information on how PM technology has now been made to work also for animal cells, and showed several approaches to how PM technology might be used to study the interaction between bacteria and animal cells. Two hot areas of research are animal cells with pathogenic bacteria growing inside, and studies of the interaction of pathogenic bacteria or probiotic bacteria with cells that line the gut. Manal Abuoun (VLA, Weybridge, UK) described how she is using PM technology to study a porcine jejunal cell line in terms of its nutrient requirements and sensitivity to ion stress, cytokines, hormones and other metabolic effectors. She intends to also look at ileal and colon cells and how *Salmonella enterica* serovar Typhimurium (*S*. Typhimurium) interacts with them.

Another presentation by Tyrrell Conway (University of Oklahoma, Norman) used PM technology to examine how *E. coli* changes its metabolism to utilize mixtures of nutrients when it grows in the mucus that lines the colon. Rather than occupying one or a few nutritionally defined niches, *E. coli* actually occupies several niches. However, not all strains occupy all available niches. *E. coli* strains differ in their *in vivo* sugar preferences, but not in their *in vitro* preferences. Furthermore, the nutrient utilization programmes of pathogenic and commensal *E. coli* strains differ substantially, which suggests that the success of pathogenic *E. coli* depends on their ability to compete for niches that may or may not be made available to them depending on the metabolism of commensal *E. coli* strains (Fabich *et al*., 2008).

## Growth properties and other phenotypes in relation to epidemiology

The keynote speech for the conference was given by Tom Cebula (FDA, Laurel, MD). His laboratory has used PM technology to analyse large collections of strains from food-borne outbreaks. He has found phenotypic analysis to be a very useful tool in strain attribution, especially for highly clonal pathogens such as *E. coli* O157, which was the focus of his talk. Tom's laboratory first found a sucrose-positive, d-serine-negative phenotype common to most O157 strains and subsequently confirmed that a sucrose operon had inserted into the d-serine operon. Genetic analysis of this genome region showed that it was a hot spot for genetic mosaicism. Another interesting result was that the O157 strain from the summer 2006 spinach outbreak in the USA had a rare *N*-acetyl-d-galactosamine-negative phenotype, which had only been found once previously (Mukherjee *et al*., 2008).

A presentation by Jean Guard Bouldin (USDA, Athens, GA) followed a similar theme. Strains of *S. enterica* serovar Enteritidis share 99.99% genomic identity, yet they vary greatly in their pathogenic properties. Because of the high degree of identity, the genetic tools typically employed to subtype strains (DNA microarray hybridization, PFGE, ribotyping) have only detected a few polymorphisms (Morales *et al*., 2005; 2006). Phenotyping, on the other hand, has provided important new insights into the magnitude of evolution present between strains. Jean's lab has focused on two strains of the same phage type (PT13a), one being a biofilm-forming strain that is a good colonizer of chickens but does not infect eggs, and the other a biofilm-negative strain that does infect eggs. Co-infection with both subtypes causes the most serious infections and disease spread (Guard-Bouldin *et al*., 2004). Phenotyping uncovered many differences between these two strains (the egg-infecting strain is more metabolically active and more resistant to antibiotics) and provided information for selecting strains for whole genome resequencing. Using an approach that tiled multiple genomes with short primers generated against a reference genome that is publicly available from the Sanger Institute, a set of 447 putative polymorphisms was detected in a first round of mutational mapping. The database, which is updated on a periodic basis as polymorphisms are characterized, is available online at http://www.ncbi.nlm.nih.gov/genomes/static/Salmonella_SNPS.html. To date, confirmatory resequencing has located approximately 70% of the putative polymorphisms to their exact genomic locations in strains of Enteritidis that are clonally related, but that nonetheless vary in virulence potential. Within the set of over 300 polymorphisms that have been confirmed, 14 polymorphisms altered open reading frames (ORFs) of predicted proteins by introducing deletions, altering terminating codons, or causing fusions of predicted proteins. Many of these proteins have no known function in propagating disease. Another 139 predicted proteins had amino acid substitutions ranging from conservative to non-conservative substitutions. Many polymorphisms were located in intergenic regions and in ribosomal genes. Identification of small-scale evolutionary events in *Salmonella* is thus contributing new knowledge about what makes one strain more virulent than another. Interestingly, the d-serine operon was also found to be a hot spot in Enteritidis, which was first made evident by PM analysis and then later confirmed by resequencing as being due to an ORF-disrupting 10 bp deletion in the gene *dsdA*. The work of both the Cebula and Bouldin labs has shown that it can be easier, more efficient and productive to go from phenotype back to genotype, instead of starting with genomic analyses.

Muna Anjum (VLA, Weybridge, UK) also spoke on *E. coli* and *Salmonella* and genotypic and phenotypic similarities and differences among strains analysed in her laboratory. An interesting example was found in the analysis of the sequenced multidrug-resistant *Salmonella s*train DT104, which caused an epidemic that peaked around 1990. They also analysed clinical DT104 strains collected during the pre-, peak- and post-epidemic phases. Muna's group found that DT104 strains had some unique changes in metabolism of histidine and glyoxylate, compared with *S.* Typhimurium LT2. First, DT104 strains were able to utilize histidine as both carbon and nitrogen source, in contrast to LT2, which harbours a frameshift mutation in the *hutU* gene. Second, all penta-antibiotic-resistant DT104 strains and most sensitive strains were unable to metabolize glyoxylate as carbon source, probably due to loss of a chromosomal region harbouring genes known to be involved in its metabolism. One DT104 antibiotic-sensitive strain that retains this region was able to utilize glyoxylate as carbon source. Muna's group is currently investigating the significance of the loss of this region in most DT104 clinical strains to determine whether it is part of a step-wise adaptation that DT104 strains have undergone to increase their virulence and survival within the human host.

Other food pathogen talks were given by Carol Iversen (University College, Dublin) and Atin Datta (FDA, Laurel, MD). Carol and her collaborators used both phenotyping and DNA analyses to update the taxonomy of the species commonly known as *Enterobacter sakazakii*. This is an important pathogen in the infant formula industry because it has caused infant mortalities. The bacterium is occasionally found in milk powders and is desiccation-resistant. *E. sakazakii* has been reclassified into a new genus, called *Cronobacter*, which contains *Cronobacter sakazakii* and four other named species: *C. malonaticus*, *C. muytjensii*, *C. turicensis* and *C. dublinensis* (Iversen *et al*., 2008). Most clinically isolated strains in Carol's collection were found to be dextrin-positive and somewhat less salt-tolerant than environmental isolates, indicating possible pathoadaptation as these organisms move from environmental to clinical niches.

*Listeria monocytogenes* is another pathogen that is problematic for the dairy industry. Although most major listeriosis outbreaks are characterized by septicaemia, meningitis, abortion and death (invasive listeriosis), several human listeriosis outbreaks were reported to have febrile gastroenteritis as the only symptom. Atin Datta and colleagues analysed a large collection of strains and found differences in phenotypic characteristics between invasive and gastroenteritis strains. Whereas the *L. monocytogenes* strains involved in invasive listeriosis outbreaks seem to be more acid-tolerant, the strains involved in gastroenteritis outbreaks appear to be more osmo-tolerant. As invasive *Listeria* infection requires survival in the gastric and phagolysomal environment, increased acid tolerance would be an advantage for invasive strains over the gastroenteritis strains, which exert their effects only in the gastrointestinal tract. Acid tolerance of *L. monocytogenes* is often correlated with the infectious dose and several studies have indicated that gastroenteritis outbreaks are always associated with the consumption of much higher numbers of *L. monocytogenes* than the invasive outbreaks. The increased osmo-tolerant nature of the gastroenteritis strains might indicate their increased ability to survive in environments including different foods.

## Biofilm formation and population ecology

Two presentations at the Conference used Phenotype MicroArrays as an experimental tool to probe the effects of culture conditions on microbes. Alex Boehm (University of Basel) screened an *E. coli* strain for its ability to form biofilms in the wells of the MicroArray and made the surprising observation that, at sublethal concentrations, translation-inhibiting antibiotics (tetracyclines, aminoglycoside, amphenicols and macrolides) trigger biofilm formation. Subsequent genetic analysis revealed that biofilm induction by translation inhibitors is mediated by the synergistic action of two small signalling molecules: c-di-GMP and ppGpp. While c-di-GMP produced by a specific diguanylate cyclase (DgcH) has a stimulatory effect on biofilm formation, ppGpp has an inhibitory effect on biofilm formation. The following model was presented: translational interference by low doses of translation inhibitors leads to a SpoT-mediated decrease of the cellular ppGpp pool and a concomitant DgcH-mediated increase of the c-di-GMP pool. Readjustment of the levels of these two signalling molecules orchestrates biofilm formation by largely unknown molecular mechanisms.

Patrick Venail (Université de Montpellier, France) and colleagues (Venail *et al*., 2008) used the diverse culture conditions of the Biolog microplates to allow a strain of *Pseudomonas fluorescens* to evolve for ∼500 generations with varying dispersal strategies. They showed that limited dispersal leads to the evolution of greater functional diversity and higher productivity in bacterial metacommunities.

## Transporters, toxicity, efflux pumps and reduction of inorganic chemicals

Another group of talks focused on studies of how chemicals get into and out of cells and are detoxified. Ian Paulsen (Macquarie University, Sydney) used Phenotype MicroArrays to improve the annotation of transporter genes in *Pseudomonas aeruginosa*. This was done by analysing knockout mutants of 78 presumptive transporter genes and seeing which phenotypes were altered. Twenty-seven of the 78 knockouts gave clear transporter phenotypes, and of these only 12 (44%) precisely matched the predicted annotation. In 10, a more precise annotation was obtained, and in 5 (18%), a significant re-annotation was enabled. New transporters were found for hydroxy-l-proline, *N*-acetyl-l-glutamate and histamine. The latter is the first histamine transporter to be identified.

Kunihiko Nishino (Osaka University) also used Phenotype MicroArrays with knockout mutants of nine putative efflux pumps in *Salmonella* and discovered interesting and important connections between efflux pumps, metal metabolism and pathogenicity. The *Salmonella*-specific MdsABC system was found to confer resistance to a wide range of chemicals among the 240 in the Phenotype MicroArray. The *acrD* and *mdtABC* pump knockouts showed similar chemical sensitivity profiles. Both are iron-regulated, induced under low-iron conditions, and export the siderophore enterobactin. Efflux pump knockouts also showed increased sensitivity to zinc and copper and decreased mouse pathogenicity. From these studies it is clear that efflux pumps play a broader role than just exporting toxic organic chemicals.

Two labs from Italy used Phenotype MicroArrays to analyse bacteria resistant to toxic soil and water pollutants. Stefano Fedi (Università di Bologna) examined a *Pseudomonas pseudoalcaligenes* that is resistant to and can degrade PCBs. Culture conditions that encouraged biofilm formation produced cells with increased resistance to PCBs. The *cheA* gene, which is necessary for biofilm formation, also alters some metabolic pathway activities. Enrico Tatti (Università degli Studi di Firenze) used Phenotype MicroArrays to examine the mechanism of Cr (VI) resistance in naturally occurring and mutant strains of *Pseudomonas corrugata* (Viti *et al*., 2007). He found genes and pathways that contribute to multiple mechanisms of chromate resistance. The mechanisms involve a sulphur starvation response, cellular supply of NADPH and DNA repair systems.

Terry Hazen (Lawrence Berkeley National Laboratory) gave the sole Conference talk on anaerobic bacteria. His laboratory has optimized Phenotype MicroArrays for the analysis of *Desulfovibrio vulgaris*, which is an important microorganism in the environment by virtue of its ability to reduce sulphate. Terry described the techniques and computer software developed in his laboratory to optimize culture media, measure growth rates and minimum inhibitory concentrations, and determine stress and co-culture syntrophy. Not only does *D. vulgaris* require anaerobic incubation, but it also produces high levels of H_2_S gas from sulphate reduction. This corrosive gas could have serious detrimental effects on the electronics in the Biolog OmniLog instrument used to record the kinetics of redox changes in Phenotype MicroArrays. Ultimately, they found the best approach was to seal the microplates inside of commercially available plastic bags with good gas barrier properties. With this method, anaerobic conditions are maintained for at least 72 h in the OmniLog. For more fastidious anaerobes, it was found that an anaerobic CO_2_-generating sachet could be placed inside the bag to maintain more stringent anaerobic conditions. For measuring redox activity of *D. vulgaris*, Hazen and colleagues actually took advantage of the H_2_S production. By including iron in the inoculating fluid, black ferric sulphide is formed and this served as a colorimetric measure of redox generation in place of the tetrazolium dye that is normally used. Several publications on the metabolism and biology of *D. vulgaris* have resulted from this work (He *et al*., 2006; Mukhopadhyay *et al*., 2006; 2007; Stolyar *et al*., 2007).

## Photobiology and fungal biology

Irina Druzhinina (Vienna University of Technology) has used Phenotype MicroArrays to analyse the carbon metabolism pathways and regulation in industrially important fungi. Plant pathogenic *Fusarium* and the mycoparasitic and cellulose-degrading fungus *Trichoderma* have been the principal fungi studied (Druzhinina *et al*., 2006; Seidl *et al*., 2006; Nagy *et al*., 2007). A particularly creative and exciting aspect of this work has been to study photobiology. Irina and colleagues systematically examined photostimulation of growth on certain carbon sources in *Trichoderma atroviride*, which is used in the biological control of plant pathogenic fungi. These studies revealed cross-talk between effects of illumination, metabolism of cyclic AMP and response to oxidative stress. In addition, her laboratory has shown that, contrary to dogma, light plays a much less important role in conidiation than does carbon source, which is the dominant determining factor (Friedl *et al*., 2008a; 2008b). Irina also exploited the versatility of Phenotype MicroArrays, using it as a tool to test for strain degeneration, to characterize DNA transformants, to search for enzyme inducers and to identify traits useful in screening of industrial fungi.

## Systems biology

Systems biology seeks to model the cell, as closely as possible, in its entirety. Modellers must rely on data available to them. Current definition and annotation of genes is good but still needs a lot of improvement, as evidenced by the data on transporter annotation presented by Ian Paulsen (see above). Understanding the regulation of genes in the context of the biology of the cell is an even greater challenge. This is being addressed somewhat with mRNA measurements using genechips, but our understanding of gene regulation is still in its infancy. There are also major limitations to what can be concluded from mRNA measurements, because they do not tell us if the protein product is translated at a corresponding level and is active *in vivo*. We need *in vivo* biological measurements to provide that information, and Phenotype MicroArrays can provide some of it. In the last year there have been six publications on bacterial systems in which Phenotype MicroArray data have been used to check on and improve models (Covert *et al*., 2004; Feist *et al*., 2007; Jones *et al*., 2007; Mols *et al*., 2007; Oh *et al*., 2007; Oberhardt *et al*., 2008).

One of the groups doing this is Krishna Mahadevan's laboratory (University of Toronto) and Laurence Yang from that group presented a review of their work. They have developed a method for reconciling growth profiling data with genome-scale models and have used this method to greatly improve their models of *Bacillus subtilis* and *E. coli*. In work on *B. subtilis*, performed in collaboration with colleagues at University of California at San Diego and Genomatica, growth rates of this bacterium were computed with a predictive model and then compared with metabolic rate information from Phenotype MicroArray assays (Oh *et al*., 2007). Of 270 cases tested, the correct qualitative prediction rate was only 53%, but this improved to 79% after assimilating the phenotypic data and adding 84 reactions to the model. To test the new model further, predictions of the growth phenotypes of knockout strains were tested and found to be accurate in 725 of 772 (94%) cases. Overall the phenotypic data revealed the requirement for 89 specific enzymes that had not been annotated and the identification of 13 genes that could be putatively assigned to enzymes.

The largest phenotyping project so far has been undertaken by Hirotada Mori's group (Nara Institute of Science and Technology). They have made knockouts of all essential genes of *E. coli* MG1655 and assayed 500 knockout strains. Hirotada discussed the data obtained with the 45 genes affecting central metabolism in *E. coli*. His software group has developed tools to convert the phenotypic assay data to multidimensional simplified vectors to allow modelling and predictions. This work is still ongoing. In a parallel project, the single gene knockouts were screened for growth on minimal medium and it was found that nearly all could still grow, indicating the availability of alternative metabolic pathways. Work is now in progress to make a set of double gene knockouts to provide another source of phenotypic information to test metabolic models.

## Sharing phenotyping technology in a core facility – the GENEXPRESS LAB

The last afternoon of the Conference featured demonstrations of software for analysis of phenotypic data by Jeffrey Carlson (Biolog) and Terry Hazen (Lawrence Berkeley National Laboratory). This was followed by a tour of the host university's new GENEXPRESS LAB, which serves as a core facility that is shared within the Dipartimento di Biotecnologie Agrarie (Università degli Studi di Firenze) and which collaborates with other research laboratories in Italy. It houses not only the phenotypic testing equipment, but also state-of-the-art equipment for doing genomic and gene expression molecular biology testing. The value of creating expertise and sharing the new phenotypic technology was demonstrated by the four diverse and successful projects presented at the conference by Enrico Casalone (Firenze), Enrico Tatti (Firenze), Emanuele Biondi (Firenze) and Stefano Fedi (Bologna). This is likely to become a model for other universities that see the need to expand the research capabilities of their core facilities to also include high-throughput phenotypic assays.

## Broad applications and utility of phenotype microarray analysis

The Florence Conference documented both the power of high-throughput phenotyping and the diverse areas of research where it can contribute uniquely powerful and essential data. To summarize, here is a list from the Conference:

- understanding phenotypic properties of diverse microorganisms;
- improving taxonomy and phenotypic description of species;
- understanding metabolic properties and culture requirements of microorganisms;
- understanding the biology of microorganisms that cannot be cultured axenically;
- understanding phenotypic properties of animal cells;
- studying the interaction of pathogenic microbes with animal cells that they infect;
- epidemiological fingerprinting of microbial strains;
- determining hot spots for genetic change in closely related pathogenic strains;
- finding relevant metabolic changes in epidemic strains;
- studying bacterial evolution and genetic population dynamics;
- finding culture conditions that stimulate or inhibit biofilm formation;
- studying the role of efflux pumps;
- studying mechanisms of chemical resistance and sensitivity;
- improving genome annotation;
- improving systems biology models of cells;
- studying effects of light on cells and its interaction with other culture conditions;
- studying cellulose degradation; and
- complementing information obtained from genechip experiments.
